# Bilaterally Variant Accessory Fibularis Muscle: Its Phylogenetic, Molecular and Clinical Perspective

**DOI:** 10.7759/cureus.12165

**Published:** 2020-12-19

**Authors:** Deepika Poonia, Swati Tiwari, Sabita Mishra

**Affiliations:** 1 Anatomy, Maulana Azad Medical College, New Delhi, IND

**Keywords:** subtalar joint, eversion, fibularis longus, fibularis brevis, calcaneum, fifth metatarsal, retromalleolar groove, peroneus quartus, jones fracture

## Abstract

Accessory fibularis muscle is prevalent in 2.9-21.8% of the world population. Incidentally during routine dissection of a 75-year-old male cadaver, bilaterally accessory fibularis muscle was observed. On both the sides, proximal site of attachment was same but muscle displayed different distal sites of insertion in the foot. Appearance of accessory muscle in the leg is indicative towards the ongoing phylogenetic evolution operating at the molecular level. Bio-mechanical advantage of this variant muscle is the additional support provided to the subtalar joint. Also it acts as synergist to fibularis longus and brevis during eversion of the foot. Clinically this muscle may predispose to chronic ankle pain, dislocation of peroneal tendons from retromalleolar groove and post fracture dislocation in foot. Wide range of accessory fibularis muscle has been previously reported with different nomenclature, however, existence of two different variants in same cadaver has been rarely reported. The current observation is significant for clinicians to acknowledge when evaluating and operating patients with foot disorders.

## Introduction

The lateral compartment of leg houses two muscles among which Fibularis (Peroneus) longus (FL) is the superficial muscle and Fibularis brevis (FB) is the deeper muscle. The fibers of FL originate from the head of fibula along with upper two-third of the lateral surface of shaft of fibula. Few fibers also arise from the deep fascia of leg, inter-muscular septa and lateral condyle of tibia. The tendons of FL and FB course distally in a common synovial sheath with FL located posterior to FB in a groove roofed by superior fibular retinaculum located posterior to lateral malleolus. Beyond this retinaculum the tendon of FL courses antero-inferiorly deep to inferior fibular retinaculum along the lateral surface of calcaneum and cuboid. Thereafter winds round the lateral surface of cuboid to reach the sole. In the sole, tendon of FL courses obliquely from lateral to medial side positioning itself in the fourth layer of sole. Just before the insertion tendon of FL splits into two slips - one attaches to the lateral surface of the base of first metatarsal and other attaches to the lateral surface of medial cuneiform. In contrast the tendon of FB traverses a shorter distance and ends by attaching to the tuberosity present on the lateral surface of the base of fifth metatarsal [1].

Electromyographic studies have shown the functional role of FL and FB during gait cycle. Both the muscles contract to cause eversion at ankle joint. FL acts as prime agonist to maintain longitudinal and transverse plantar arch of foot [1]. In addition, the tendons of FL and FB stabilize the ankle joint. Occasionally an accessory muscle may be found in the lateral compartment of leg. This extra slip has been reported to have beneficial as well as detrimental effects. It may be an additional source of graft harvest for tendon transfer surgeries and autograft [2-4]. Bilgili et al. mirrored the drawbacks of having a variant muscle in lateral compartment of leg by reporting statistically significant correlation between existence of accessory muscle and longitudinal degeneration of FB which may result in ankle pain [2].

The current literature encloses reports about accessory muscle in the lateral compartment of leg referred to as, “Accessory Fibularis Muscle” (AFM) or “Peroneus Quartus” with variable proximal and distal sites of attachments. We report a unique case with AFM observed bilaterally both exhibited variable sites of insertion. Along with clinical significance, we have also addressed the embryological and molecular basis of this serendipitous muscle, which was not previously emphasized. Acknowledgment of this variant anatomy is paramount to distinguish one accessory muscle from other muscles in the same compartment on MRI and ultrasound scans [5]. Awareness of this variant finding will definitely facilitate clinicians to make differential diagnosis in patients attending outpatient departments with chronic persistent ankle pain and swelling.

## Case presentation

**Figure 1:**
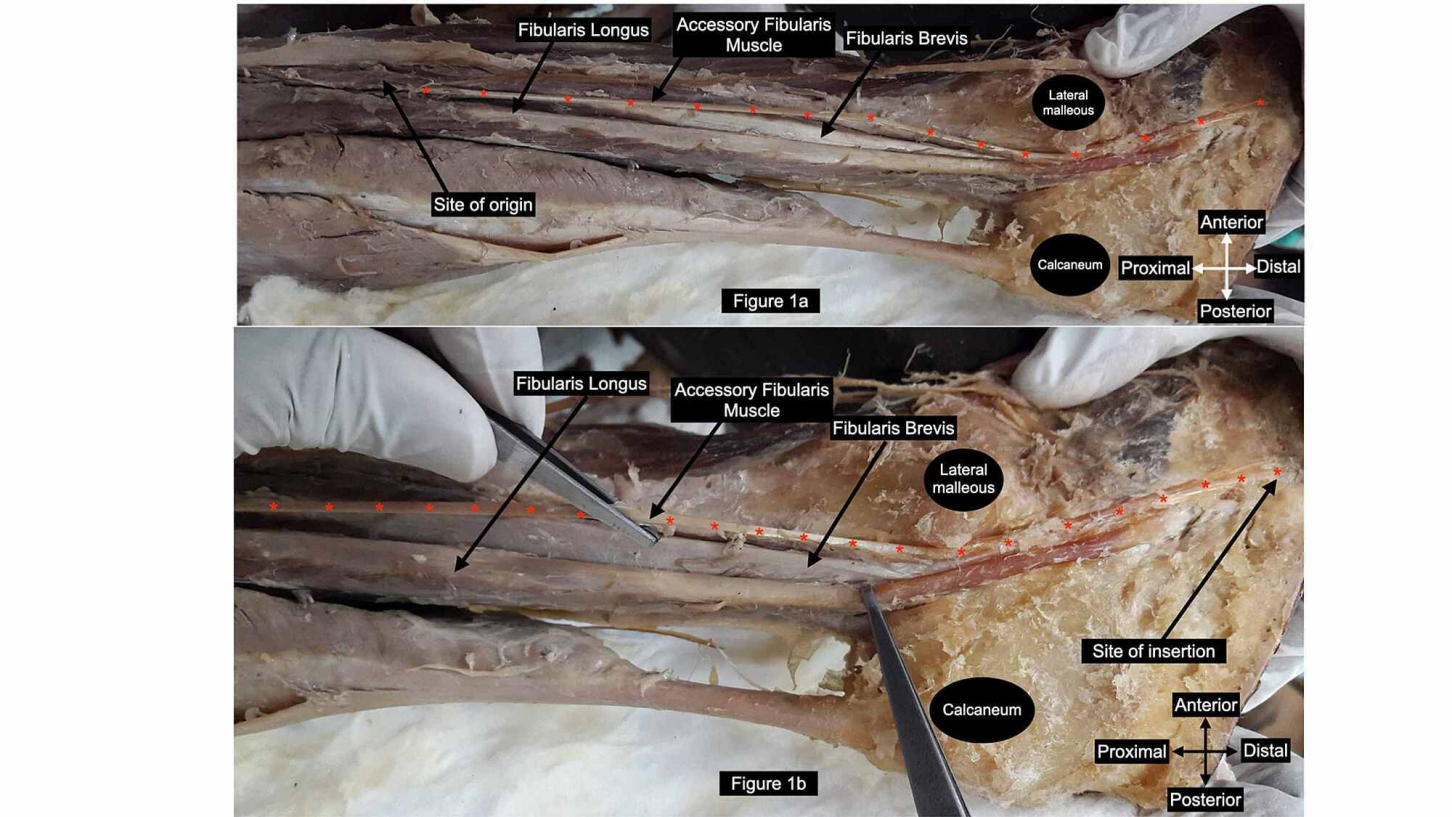
Right leg with accessory fibularis muscle showing proximal site of attachment on fibula in conjunction with fibularis longus; distal site of attachment on fifth metatarsal.

**Figure 2:**
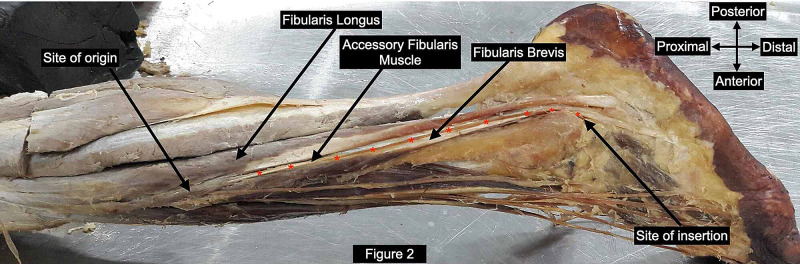
Left leg with accessory fibularis muscle showing proximal site of attachment on fibula in conjunction with fibularis longus and distal site of attachment on calcaneum.

During routine undergraduate teaching session dissection of the lower limb of a 75-year-old formalin embalmed male cadaver was performed. This work was done on voluntarily donated body to the Department of Anatomy for teaching and research purposes. FL and FB muscles were delineated from their site of origin on fibular shaft till their site of insertion in the foot. We noticed an additional muscle present bilaterally in the postero-lateral compartment of the leg. The site of origin and musculo-tendinous architecture of this variant muscle was similar on both the sides. However, site of insertion differed. Bilaterally the variant muscle originated from the upper one-third of the lateral surface of the shaft of fibula. The upper fibers of FL were seen to arise commonly with this additional muscle. This additional muscle has been referred to as “Accessory Fibularis Muscle” (AFM). The accessory muscle exhibited short muscle belly (6 cm long on right and 8.5 cm on left) and long tendinous part (37.0 cm long on right and 32.2 cm on left) which was topographically related superficial to FB. The tendon of accessory muscle (Figures 1, 2) coursed distally deep to superior and inferior peroneal retinaculum along with FL and FB.

Further on the right side the tendon coursed antero-inferior in the foot to attach to the lateral surface of base of fifth metatarsal in conjunction with the FB (Figure 1a, 1b). Hence it acted as a musculo-tendinous bridge between FL and FB. On the left side the tendon ended by attaching to the lateral surface of calcaneus (Figure 2). Bilaterally accessory fibular muscle was innervated by superficial peroneal nerve and anterior tibial artery.

## Discussion

We report a novel case with bilateral supernumerary muscle which took origin from upper part of lateral fibular surface normally occupied by FL. The site of insertion was varied in left and right foot. To the best of our knowledge this is among the rare cadaveric reports underscoring the variant muscle present bilaterally with different distal sites of attachment. Previously Clarkson et al. reported bilaterally vivid variations in same cadaver, however, they documented the site of insertion of variant muscle on cuboid on one side and calcaneum along with few slips to cuboid on other side [6]. Surgeons and radiologists must be aware about the possibility of bilateral presence of variant muscle during making provisional diagnosis of chronic ankle pain.

The accessory fibular muscle (AFM) was first explained by Otto (1816). Later Hecker studied the muscle in detail and reported the existence of accessory muscle in 13% of the cadaveric specimens [7]. According to the existing scientific library text, prevalence of AFM ranges from 2.9 to 21.8% [3-5,7,8]. However, a cadaveric study on 70 Indian specimens estimated much lower prevalence in Indian population (4.3%) [3]. The reported prevalence of bilateral occurrence of accessory fibularis muscle is 0.36% [6]. Cheung reported the prevalence of AFM higher in male which is often bilateral and unilateral in females [4]. Researchers have categorized AFM on the basis of origin and insertion as peroneus quartus [2,6,8-10], peroneus accessorius [10], peroneus digiti minimi (quinti brevis) [2,8,10], peroneocalcaneus externum, peroneocuboideus, peroneocalcaneocuboideus [7], etc. We report a novel variation which distally resembles peroneus digits minimi on the right and peroneocalcaneus on the left side, however, unlike these named variants the current variation originated from upper part of shaft of fibula in conjunction with FL hence can be called as, “Fibulodigitiminimi” on right and “Fibulocalcaneus” on left side.

Embryological and molecular basis

Limb musculature in embryonic life develops from para-axial mesoderm which undergoes segmentation to form somites. During 5th week myotome derived from somites begin to migrate into the limb buds where they form two condensed masses - ventral and dorsal muscle mass. Later the dorsal condensation differentiates to form fibularis (peroneus) longus. Molecular basis of delamination followed by migration of myogenic cells involves Pax3, c-Met, Hgf and Lbx1 gene. Myogenic cells expressing c-Met, under the influence of Hgf signalling undergo delamination and begin to migrate towards the limb buds. Thus regulation of c-Met and Hgf receptors by Pax3 is paramount for myogenic cell migration. After reaching the limb bud, myogenic cells multiply in presence of Meox2/Mox2 (a homeobox gene). Deletion of Meox2 results in absence or variation in the morphology of limb muscle [11]. McMurrich postulated that longitudinal split of muscle primordia results in the formation of two different muscles with common nerve supply [12]. As depicted by the site of origin of AFM in continuation with that of FL it can be proposed that, the accessory muscle in present report developed by splitting of muscles from FL.

Phylogenetic basis

Hecker reported the absence of AFM in prosimian and simian population and correlated the sporadic appearance of this muscle in human population with the acquisition of bipedal gaits. Thus Hecker postulated that, existence of AFM in human population is an indication of ongoing progressive phylogenetic evolution. Additionally he stipulated the role of AFM in evolution of bipedal gaits and stabilisation of subtalar joint during pronation and supination [7]. Thereby, suggested ongoing epigenetic change in humans.

Currently published literature encloses vivid data about variations in fibular group of muscles [6,13-15]. Sobel et al. conducted foetal study and classified AFM into two types - Type A (Fibularis quartus) and Type B (Fibulocalcaneus externus) [13]. We observed Type B variant in the left leg of the cadaver. Jayakumari et al. reported a case of AFM which distally merged with the tendon of FL just above the lateral malleolus [14]. Unlike Jayakumari et al. we report an accessory muscle which merged with the FB at its distal site of attachment on fifth metatarsal.

Furthermore literature also includes clinical reports encompassing serendipitous detection of accessory muscle in the lateral compartment of leg in patients complaining lateral retromalleolar ankle pain and swelling [15,16]. It was observed that topography of AFM renders nearby structures like FB to undergo attrition due to pressure atrophy under peroneal retinaculum or FL and FB to subluxate [17] or may itself get ruptured due to undue trauma [4,15]. Hence cases with chronic lateral ankle pain must be checked for the presence of AFM [2,6]. Besides, the variant muscle inserting on fifth metatarsal may predispose to fracture dislocation in cases with Jones fracture by exerting stress on fractured bone and hence may delay the bone healing [6]. Moreover on cross-sectional imaging this supernumerary muscle may resemble a mass lesion resulting in misinterpretation of normal scan as abnormal [6].

## Conclusions

Considering the propensity to generate clinically significant complications we infer that reporting similar variations is paramount as it will facilitate clinicians to make correct diagnosis on MRI, ultrasound scan and during physical examination of patients with lateral ankle pain and swelling. Also these reports will help biologists to understand the epigenesis behind ongoing phylogenetic development in human population.
